# Se-doping dependence of the transport properties in CBE-grown InAs nanowire field effect transistors

**DOI:** 10.1186/1556-276X-7-159

**Published:** 2012-02-28

**Authors:** Leonardo Viti, Miriam S Vitiello, Daniele Ercolani, Lucia Sorba, Alessandro Tredicucci

**Affiliations:** 1 NEST, Istituto Nanoscienze-Consiglio Nazionale delle Ricerche (CNR) and Scuola Normale Superiore, Piazza San Silvestro 12, Pisa, 56127, Italy

**Keywords:** nanowires, Se-doping, field effect transistors, transport, InAs

## Abstract

We investigated the transport properties of lateral gate field effect transistors (FET) that have been realized by employing, as active elements, (111) B-oriented InAs nanowires grown by chemical beam epitaxy with different Se-doping concentrations. On the basis of electrical measurements, it was found that the carrier mobility increases from 10^3 ^to 10^4 ^cm^2^/(V × sec) by varying the ditertiarybutyl selenide (DtBSe) precursor line pressure from 0 to 0.4 Torr, leading to an increase of the carrier density in the transistor channel of more than two orders of magnitude. By keeping the DtBSe line pressure at 0.1 Torr, the carrier density in the nanowire channel measures ≈ 5 × 10^17 ^cm^-3 ^ensuring the best peak transconductances (> 100 mS/m) together with very low resistivity values (70 Ω × μm) and capacitances in the attofarad range. These results are particularly relevant for further optimization of the nanowire-FET terahertz detectors recently demonstrated.

**PACS: **73.63.-b, 81.07.Gf, 85.35.-p

## Introduction

The growth of semiconductor nanowires (NWs) has recently opened new paths to silicon integration in device families such as light emitting diodes, field effect transistors (FET), high-efficiency photovoltaics, or high-responsivity photodetectors [1-3]. However, to bring the nanowire technology to a significant level of maturity and direct impact on microelectronic and photonic device applications, a more detailed level of control of the nanowire physical properties, material morphology, size, and composition is needed.

Semiconductor nanowires are conventionally grown from metallic seed particles, meaning that the mechanisms for impurity incorporation are different from those of other growth techniques [4]. The most significant difference is related with the evidence that in nanowire growth, the surfaces of already grown segments are exposed to impurities for the duration of the growth, which can affect the physical and chemical properties of such segments.

In a 'bottom-up' vision, semiconductor nanowires can be considered as peculiar 'building blocks', easily synthesizable as an active element for high performance electronic devices, whose specific characteristics are highly dependent on the nanowire physical properties. In this perspective, InAs can be identified as one of the most successful candidates for such nanoscale integration. InAs nanowires have indeed small electron effective mass and correspondingly high bulk electron mobility [4] that is typically limited by surface scattering, unless suitable core-shell heterostructure is adopted to avoid the impact of surface states [5]. Moreover, InAs nanowires can be grown epitaxially on silicon without the use of gold seeding [6], thus making the process viable also for low-cost silicon technology integration where deep Au levels in the Si bandgap must be avoided.

The potentially long electron mean free path peculiar of the III-V semiconductor materials makes InAs nanowires ideal for the fabrication of high electron mobility transistors or field effect transistors since high transconductance (*g*), at very low drive voltages, can be more easily obtained. In addition, the narrow InAs bandgap and degenerated Fermi-level pinning further allow for easy formation of excellent ohmic contacts, the latter aspect being increasingly important as the transistor dimension is scaled down. However, one of the actual potential roadblocks for the development of a robust nanowire-based FET technology is mantaining a high on-current when downscaling the nanowire diameter. The latter must indeed follow the scaling of the gate length due to electrostatic considerations, which leads to a rapid increase in the series resistance of the source and drain regions [7]. Several approaches can be followed to reduce this series resistance: metal diffusion [7], deposition of a highly conductive material around the wire [8], or doping. In particular, n-type nanowire doping can be used both to control the charge density, optimizing source-drain and contact resistance, and to ensure sharp 'pinch-off' in the transconductance.

Selenium incorporation has proven a valuable solution to achieve n-doping of InAs nanowires grown from metal organic precursors [4]. While very detailed studies have recently been published for metal organic vapor phase epitaxy, grown nanowires through capacitance-voltage characterization [9], a systematic analysis of chemical beam epitaxy (CBE)-grown structures, is still lacking.

In this work, we report a thorough investigation of the structural and transport properties of Selenium-doped, CBE-grown InAs nanowires with varying doping concentration. For the purpose, we employ simple lateral gate low-capacitance FET geometry, which despite not being ideal for the best transistor characteristics, has allowed the realization of the first plasma wave detectors operating at terahertz frequencies, also thanks to the typical attofarad-order capacitance [10].

## Experimental methods

InAs nanowires, with diameters in the range 30 to 80 nm and lengths of about 2 to 3 μm, have been grown by CBE in a Riber Compact-21 system (RIBER MBE, Paris, France) by Au-assisted growth [10,11] on InAs (111) B substrates. Trimethylindium (TMIn) and tertiarybutylarsine (TBAs) were employed as metal organic (MO) precursors for InAs growth [11] and ditertiarybutyl selenide (DtBSe) as a selenium source for n-type doping. Due to its high decomposition temperature, TBAs was pre-cracked in the injector at 1000°C. A nominally 0.5 nm thick Au film was first deposited by thermal evaporation on the InAs wafer in a separate evaporator chamber and then samples were transferred to the CBE system. The wafer was then annealed at 520°C under TBAs flow in order to remove the surface oxide and generate the Au nanoparticles by thermal dewetting. The InAs segment was grown for 90 min at a temperature of (430 ± 10)°C, with MO line pressures of 0.3 and 1.0 Torr for TMIn and TBAs, respectively. For n-type doping, the DtBSe line pressure was adjusted from 0 to 0.4 Torr to achieve different carrier densities [4].

**Figure 1:**
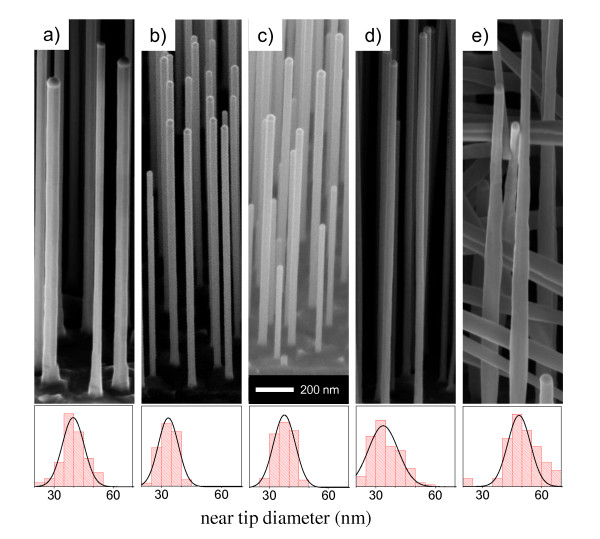
**(Samples a, b, c, d, and e) 45° tilted SEM image of the investigated InAs nanowires**. They were 1 to 2 μm long and grown with DtBSe precursor line pressures of (a) 0 Torr; (b) 0.05 Torr; (c) 0.1 Torr; (d) 0.2 Torr; and (e) 0.4 Torr. The bottom panels show the histograms representing the as-grown nanowire near-tip diameter distribution of the corresponding NW samples. The solid line is a Gaussian fit of the distribution.

Figure 1 shows the scanning electron microscopy (SEM) images of the as-grown NWs standing on the InAs substrate recorded by using a field emission SEM operating at an accelerating voltage of 5 kV, while keeping the wires tilted at a 45°angle. Five individual nanowire growths have been performed by increasing the DtBSe line pressure from 0 (sample a) to 0.4 Torr (sample e) with intermediate values of 0.05 Torr (sample b), 0.1 Torr (sample c) and 0.2 Torr (sample d). The corresponding SEM images are reported in the panels (Figure 1 samples a, b, c, d, and e), respectively. The statistical distributions of the nanowire (NW) near-tip diameter are reported on the bottom panels of each SEM image.

In all cases, the In-Au catalyst particle is clearly visible at the tip of each NW. Moreover, apart from the most highly doped sample, all NWs are untapered and show no sign of lateral or irregular growth (Figure 1 samples a, b, c, and d). In contrast, the heavily doped sample shows some lateral growth with irregular faceting indicating the onset of a different growth regime. Such lateral overgrowth in the highly Se-doped InAs NWs is correlated with the increased presence of stacking faults in Wurtzite InAs up to the limit of having large zinc-blende sections [4]. Indeed, electron diffraction experiments in low-doped samples indicate a Wurtzite-like crystal structure, while highly doped NWs also show zinc-blende segments in agreement with previous experimental reports [4].

**Figure 2:**
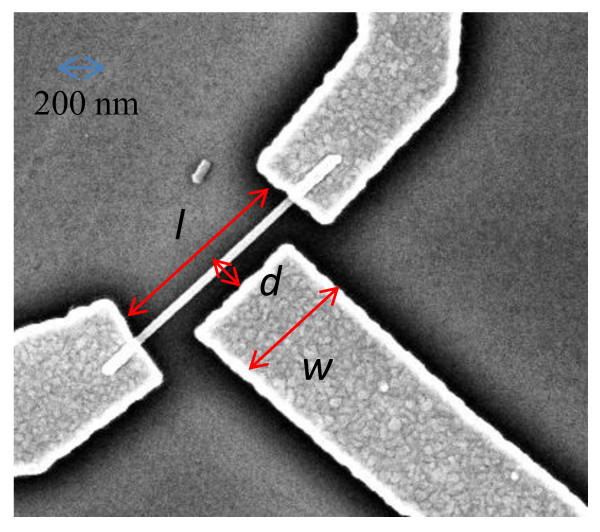
**SEM image of a field effect transistor based on an InAs nanowire**. The red arrows indicate the source-to-drain distance (*l*), the gate length (*w) *and the gate-to-nanowire distance (*d*).

The NWs have been then mechanically transferred to a 350-μm thick Si substrate with a 500 nm SiO_2 _insulating surface layer. The samples then have been spin-coated with an e-beam sensitive polymethylmethacrylate-negative tone resist, and electron beam lithography has been used to define the transistor source, drain, and gate nanoscopic contacts linking individual nanowires to the macroscopic contact pads. InAs NWs grown with different DtBSe fluxes have been employed to lithographically define field effect transistors having comparable channel length (*l *= 1,200 to 1,300 nm) in a lateral gate configuration (Figure 2) with similar gate lengths (*w = *750 to 850 nm) and distances (*d *= 120 to 130 nm). A detailed summary on the fabricated devices with their average geometric dimensions and the corresponding NW diameter is reported in Table 1.

**Table 1:** Samples

Sample	Metal organic line pressure (Torr)	Nanowire diameter (nm)	Gate-to-nanowire distance (nm)	Gate length (nm)	Source-to-drain distance (nm)
a	0	29	120	750	1,200

b	0.05	33	140	700	1,200

c	0.1	39	140	750	1,250

d	0.2	40	160	870	1,400

e	0.4	80	140	1,700	2,500

Average geometrical values for FET devices based on InAs nanowires having different diameters and grown under different DtBSe precursor line pressures. The NW diameters have been measured at the center of the NW axis.

After the development of the exposed resist, residual polymer on the nanowire contact areas was removed using mild oxygen plasma. Before metal contact deposition, surface oxides must be removed to ensure low resistivity ohmic contacts, and the InAs contact areas should be passivated to prevent reoxidation. A passivation step is then performed before evaporation using a highly diluted ammonium polysulfide or NH_4_S_x _solution. This treatment has proven to be crucial for an optimal electrical behavior of our devices due to the reduced contact area resulting from the high nanowire surface to volume ratio [12]. Moreover, this treatment potentially prevents the surface scattering processes that induce a significant reduction of the nanowire mobility [13,14].

Ohmic contacts have been realized by thermally evaporating a Ti(10 nm)/Au(90 nm) layer onto the samples. Samples are then imaged by SEM in order to check the processing results and then glued on a standard ceramic dual in-line package. About 30 nanowire-based FETs (6 for each individual nanowire growths), with comparable geometrical dimensions, have been realized. Each device has then been wire-bonded by thin Au wires (25 μm diameter) with a semiautomatic facility.

To proceed with the electrical characterization of the fabricated devices, we have independently driven at room temperature, in air, the source-to-drain (*V*_sd_) and the gate-to-ground (*V_g_*) voltages, in ranges [-0.025 to 0.025 V] and [*-*10 to 10 V] by using the digital to analogue converter of a lock-in amplifier with the source grounded. The drain contact has been connected to a current amplifier that converts the current into a voltage signal amplified by a factor of about 10^4 ^V/A. The latter signal has been measured with a voltmeter reader.

## Results and discussion

**Figure 3:**
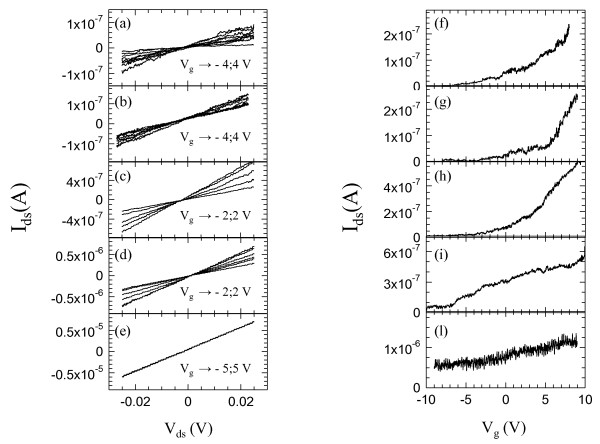
**Electrical transport characteristics of the transistors**. Patterns a, b, c, d, and e refer to current-voltage (*I*_sd_-*V*_ds_) characteristics measured at different gate voltages *V_g_*, at room temperature for samples a, b, c, d, and e, respectively; Patterns f, g, h, i, and l refer to *I*_sd_-*V*_g _transfer characteristic measured for samples a, b, c, d, and e at room temperature and at a drain-to-source voltage *V*_ds _= 0.025 V.

Figure 3 patterns a, b, c, d, and e show the source-drain current (*I*_sd_)-*V*_sd _characteristics measured at different values of *V_g _*for each prototype-investigated samples (one for each individual CBE growth of Figure 1). The samples have been labeled as a, b, c, d, and e referring to the mentioned CBE growth conditions.

By driving the lateral gate at positive voltage values, the nanowire resistance decreases by about two orders of magnitude. Moreover, regardless of the nanowire carrier density, a linear increase of the nanowire current has been observed as a function of the source-drain bias with slopes progressively larger with increasing n-doping concentration. Indeed, at a gate voltage *V_g _*= 10 V, the nanowire resistance (*R*) varies from 470 to 3 kΩ while the DtBSe line pressure is increased from 0 to 0.4 Torr. By approximating the nanowire with a cylindrical geometry, we calculated the nanowire resistivity *ρ *as *ρ *= *RπD*^2^*/*4*l*, where the nanowire diameter *D *has been obtained by averaging the diameter of the nanowire over its free length *l*, between the contacts. Resistivity values in the range of 300 to 8 Ω × μm have been measured for nanowires grown with increasing precursor line pressure.

The carrier density has also a dominant role on the FET 'gating effect' that is based on the manipulation of the charge distribution. The applied gate bias indeed modifies the charge distribution in the nanowire, namely, the electron carrier density in the n-channel of the FET.

Figure 3 patterns f, g, h, i and l show the change of the *I*_sd _as a function of the gate voltage. A gate voltage sweep has been applied to the nanowire transistor by keeping a fixed *V*_sd _= 0.025 V value. In all cases, at negative gate voltages, the electric field is able to produce a depletion region narrowing the channel and turning off the current flowing through the nanowire when *V_g _*approaches the pinch-off voltage value. In the last case (Figure 3 patterns e and l), the gate potential is indeed not able to remove the electrons and a current still flows through the channel even at *V_g _*= *-*10 V.

**Figure 4:**
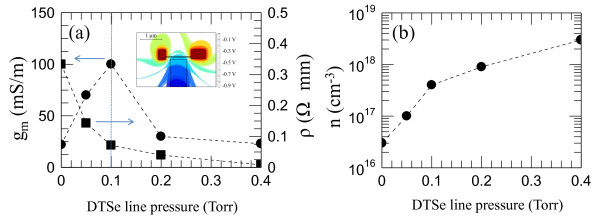
**Peak transconductance and extracted carrier density**. (**a**) Peak transconductance (black circles) normalized to the gate length and resistivity (black squares). Values are plotted as a function of the DtBSe precursor line pressure. Inset: 3D finite element electrostatic potential simulation calculated by applying a gate voltage *V*_g _= -1 V at sample c while keeping *V*_ds _= 0. Continuous boundary conditions are applied to the air-Si interface. Isosurfaces corresponding to different values of the electrostatic potential are shown on the graph. (**b**) Extracted carrier density (black circles) in the transistor channel as a function of the DtBSe precursor line pressure.

The threshold voltages *V*_th _of our nanowire FETs have been determined by the intercepts with the horizontal axis of the linear fit of the *I*_sd_-*V_g _*characteristics in the region of maximum transconductance (*g_m_*). The comparison between Figure 3 patterns f, g, h, i and l show that *V*_th _progressively decreases from -30 to -2.5 V as a function of the precursor pressure, i.e., by increasing the carrier density. However, the analysis of the extracted slopes shows that the peak transconductance, normalized to the gate length, varies from 10 to 100 mS/m reaching to a maximum when the precursor pressure of 0.1 Torr is employed (Figure 4a). The strong increase of the Se doping suppresses the peak transconductance, probably due to the screening of the gate by holes or the more ionized donors for electrons to scatter from.

Under the same experimental conditions, a minimum inverse subthreshold slope of ≈ 11 V/dec has been found. Figure 4a shows the change of the transconductance and resistivity values measured for samples a, b, c, d, and e as a function of the growth conditions. The comparison between the *g_m _*curve and the corresponding *ρ *values shows that to optimize the transport properties of the FETs, a compromise should be found between high transconductances/low threshold voltages and sufficiently low nanowire resistivities. By using the measured transconductances, we can extract the carrier mobility (*μ*) in our nanowires by means of the Wunnicke metrics [15]. The latter correlates the gate capacitance *C *to nanowires deposited on top of SiO_2 _with the carrier mobility through the equation *μ *= *g_m_l*^2^*/(CV*_ds_*)*. By using a 3D finite element simulation of the electrostatic potential (inset of Figure 4a), a gate capacitance value of *C *= 2.8 aF has been estimated at *V_g _*= 0 V while keeping *V*_ds _= 0.025 V. Mobility values in the range [10^3 ^- 10^4^] cm^2^/(V × sec) have been extracted for samples [a-e]. From the mobility and resistivity data, we extracted the carrier concentration, *n*, at *V_g _*= 0 V from the relation *n *= (*ρeμ*)^-1^. This allows us to correlate the precursor line pressure during Se doping with the effective carrier density through the nanowire.

The results plotted in Figure 4b show that the carrier density in our devices increases significantly from DtBSe line pressures above 0.1 Torr, and that the best compromise in the FET transport performances (dashed line in Figure 4a) is found at *n *= 5 × 10^17 ^cm^-3^

**Figure 5:**
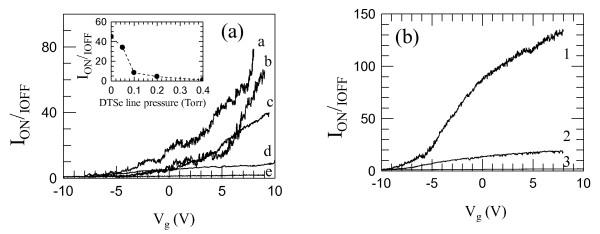
**On/off current ratio *I*_on_/*I*_off _as a function of the gate bias**. Panel (**a**) refers to the investigated set of samples a, b, c, d, and e. Inset: maximum value of the on/off current ratio plotted as a function of the DtBSe precursor line pressure. Panel (**b**) was measured for FET devices having identical growth conditions and geometries of sample c with gate-to-nanowire distances of 290 (curve 3), 125 (curve 2), and 70 nm (curve 1), respectively.

To quantitatively compare the *I*_sd_*-V*_*g *_transfer characteristics of our FETs, regardless of their specific resistances, we extracted the on/off current ratio *I*_on_*/I*_off _as a function of the gate bias, where *I*_on _corresponds to *I*_sd _while *I*_off _is the off-state diffusion current through the channel at reverse gate bias *V_g _*<*V*_th _[16]. The results are plotted in Figure 5a.

Note that the employed definition of on/off current ratio differs from the conventional meaning for depletion mode transistors in nanoelectronics. The latter choice is mostly due to the fact that, in the present case, the conventional definition of *I*_on _as *I*_sd _at *V*_sd _*= V_g_-V*_th _would hinder a quantitative comparison of the performance of our devices, since very low values of the applied source-drain voltage must be used in order not to damage the devices.

Despite the higher normalized peak transconductance measured in sample c, the latter device did not show the maximum *I*_on_*/I*_off _ratio, as evident from the inset of Figure 5a. This is mostly due to the very low *I*_off _current measured only in low-doped devices. In the latter case, the electric potential induced by the gate electrode is capable of removing all the carriers from the channel. On the contrary for heavily doped samples c, d, e, the latter condition is not verified and *I*_off _is always larger than 0.2 μA even at *V_g _*= -10 V.

We finally compared the measured *g_m _*and subthreshold slope values with those reported in the literature [17] for wrap-gated InAs nanowires. For our best sample, a maximum *g_m _*value about three orders of magnitude smaller and a subthreshold slope two orders of magnitude larger have been found. This discrepancy can be addressed to the less favorable device geometry. To quantify the observed discrepancy, we performed 3D finite element simulations of the electrostatic potential. An example is schematically pictured in the inset of Figure 4a. The employed lateral gate configuration leads to gate-wire capacitance values of ≈ 3 aF, whereas in the wrap-gate geometry, this capacitance is about 0.1 fF, thus ensuring a stronger coupling between the gate and the transistor channel. As a possible way to increase such coupling, we engineered lateral gates located at progressively reduced gate-nanowire distances. We processed a set of nanowire FETs by using the nanowire growth conditions employed for Figure 1 sample c by maintaining identical contact geometries and while varying the parameter *d*. Figure 5b shows the comparison of the *I*_on_*/I*_off_-*V_g _*characteristics measured as a function of the parameter *d*. By decreasing *d *by a factor of 3, the nanowire capacitance increases up to 4 aF, and the normalized peak transconductance reaches a maximum value of 380 mS/m i.e., ≈ four times larger than that measured for Figure 1 sample c.

## Conclusions

The electrical properties of FETs realized by employing (111) B-oriented InAs nanowires with different carrier densities as active elements were studied. The presented data show that the doping leads to a significant decrease in the relative values of the resistivity. We found that by keeping the DtBSe precursor line pressures at 0.1 Torr, the carrier density in the nanowire channel increases up to 5 × 10^17 ^cm^-3 ^assuring good peak transconductances (> 100 mS/m) and very low resistivity values (70 Ω × μm) ideal for the development of high performance nanoelectronic and photonic devices [10]. Further improvements can be obtained by increasing the coupling of the gate-nanowire elements and/or by optimizing the chemical passivation thus reducing scattering processes at the nanowire surface [18], or implementing epitaxial core-shell growth processes [5].

## Competing interests

The authors declare that they have no competing interests.

## Authors' contributions

LV carried out the device fabrication and testing; DE and LS carried out the nanowire growth and structural characterization; MSV contribute to the device fabrication and testing, conceived the study and wrote the manuscript; AT conceived the study and participated in its design. All authors read and approved the final manuscript.
